# Selfish routing equilibrium in stochastic traffic network: A probability-dominant description

**DOI:** 10.1371/journal.pone.0183135

**Published:** 2017-08-22

**Authors:** Wenyi Zhang, Zhengbing He, Wei Guan, Rui Ma

**Affiliations:** 1 MOE Key Laboratory of Urban Transportation Complex System Theory and Technology, Beijing Jiaotong University, Beijing, P. R. China; 2 Department of Civil and Environmental Engineering, University of California, Davis, California, United States of America; Beihang University, CHINA

## Abstract

This paper suggests a probability-dominant user equilibrium (PdUE) model to describe the selfish routing equilibrium in a stochastic traffic network. At PdUE, travel demands are only assigned to the most dominant routes in the same origin-destination pair. A probability-dominant rerouting dynamic model is proposed to explain the behavioral mechanism of PdUE. To facilitate applications, the logit formula of PdUE is developed, of which a well-designed route set is not indispensable and the equivalent varitional inequality formation is simple. Two routing strategies, i.e., the probability-dominant strategy (PDS) and the dominant probability strategy (DPS), are discussed through a hypothetical experiment. It is found that, whether out of insurance or striving for perfection, PDS is a better choice than DPS. For more general cases, the conducted numerical tests lead to the same conclusion. These imply that PdUE (rather than the conventional stochastic user equilibrium) is a desirable selfish routing equilibrium for a stochastic network, given that the probability distributions of travel time are available to travelers.

## 1. Introduction

The urban road network is an open network that ceaselessly receives various disturbances, such as traffic incidents, weather variation, road maintenance, special events, and temporal factors. These disturbances are usually unpredictable, which stimulate the demand for an applicable approach to describe travelers' routing behavior and to model the resulting equilibrium on a road network under uncertainty. During the past decades, a large body of literature focused on this topic, which can be generally classified into two categories, i.e., the studies on the stochastic user equilibrium (SUE) model and the studies on reliability-based user equilibrium (RUE) model. A brief literature review is given as follows along these two lines.

The SUE model, firstly shaped by Daganzo and Sheffi[1], is a well-known approach to analyze stochastic traffic network. It is assumed that the network information is unavailable and travelers choose routes according to their perceived travel time that usually consists of two additive parts, i.e., an observable term and an unobservable random error term. At SUE, travel demands are assumed to assign to each route in the effective route set by the fraction of choice probability (i.e., the probability of a route dominating the others in the same route set). When the random error terms follow the independent and identical Gumbel distribution, SUE can be formulated by the multinomial logit (MNL)[2] model. When the random error terms distribute normally, SUE can be formulated by the multinomial probit (MNP)[1] model. MNL has a closed form while MNP does not, making MNL much more convenient and thus more popular than MNP. However, the independent and identical distribution assumption brings two criticisms against MNL, i.e., inability to account for overlapping among routes and inability to account for different perceived variance. To overcome the criticisms, many extensions are suggested, and the readers are referred to Kitthamkesorn and Chen[3] for a review on these extended models. Another type of closed-form SUE model assumes that the perceived travel time distributes Weibull, where the perceived travel time is not additive but multiplicative[4]. Castillo et al.[5] developed the first model. Kitthamkesorn and Chen[3] recently proposed a so-called path-size Weibit model by taking the route correlation into account, which can defuse the above two criticisms in MNL. However, the multiplicative perceived travel time is not intuitive. In addition to developing new models, another inseparable topic is to define a reasonable route set, since the final SUE depends not only on the route choice probability but also on the route set. The former estimates how many demands will be assigned on each route, while the latter determines which routes can be assigned. Various route sets were applied in different studies[2,6–8], but, to date, there is not a widely accepted one. More comprehensive reviews with respect to the SUE models can be found in the existing studies[9–11].

In contrast, the RUE model assumes that travelers know the probability distribution of route travel time, and route choice in a stochastic network is treated as a risk decision-making, where travelers not only intend to shorten travel time but also to improve punctual arrival probability (or say arrival reliability). Thus, the essence of a RUE model is to identify a proper reliability-based measure to capture travelers' reliability aspiration. In the existing literature, the reliability-based measure is usually expressed as the summation of the mean travel time and a safety margin[12], and different definitions with respect to safety margin lead to different reliability-based measures, e.g., the percentile travel time[13], the travel time budget[14], the mean-excess travel time[15], and the combined mean travel time[16]. For a RUE model, the collective reliability-guided routing behavior in a stochastic road network is then viewed as a non-corporative game with pay-off quantified by the reliability-based measure, and the resulting routing equilibrium corresponds to RUE.

In the most recent years, with the rapid development of information technology, travel information is increasingly collectable and available[17–19]. Seeing this trend, this paper suggests a probability-dominant user equilibrium (PdUE) model to describe the selfish routing equilibrium in a stochastic traffic network where the probability distributions of route travel time are assumed to be available to travelers. The current PdUE model borrows some notions from the SUE model and redefines them accordingly. In addition, PdUE is defined in the framework of the non-corporative game. At PdUE, travel demands are only assigned to the routes with the largest dominance, and a well-designed route set is not necessary. The main contributions of this study are: i) PdUE is proposed and illustrated, and a behavioral dynamic model is developed to explain the formation mechanism; ii) The logit formula of PdUE, as well as the equivalent varitional inequality formulation, are derived and analyzed; iii) Given travel time distribution and through a hypothetical experiment, the SUE and PdUE routing strategies are discussed exhaustively from an individual selfish perspective.

The remaining text is organized as follows. The definition, the general formulation, and the behavioral dynamic description with respect to PdUE are stated in Section 2. Section 3 elaborates on the logit formula of PdUE, as well as its equivalent varitional inequality formulation. Section 4 provides an exhaustive discussion on two routing strategies. Section 5 concludes the study and suggests some future works.

## 2. Probability-dominant user equilibrium

In this section, the definition and general formulation of the present PdUE are stated first, and an intuitive rerouting dynamic model is then proposed to describe the travelers' dominance-seeking rerouting behavior.

### 2.1 Definition and formulations

Consider a strongly-connected stochastic traffic network and assume that travelers have knowledge of the route travel time distribution. Let *A* be the set of links, *a* be the link index, *W* be the set of origin-destination (OD) pairs, *w* be the OD pair index, *R*^*w*^ be the set of acyclic paths in OD pair *w*, *r* be the path index, and the route travel time be a random variable that can be additively expressed by the following two terms.

$$U_{r}^{w}=C_{r}^{w}+\varepsilon_{r}^{w}\;\forall r\in R^{w},w\in W,$$ (1)

where $U_{r}^{w}$ is the stochastic travel time of path *r* in OD pair *w*, $C_{r}^{w}$ is the measurable (or say deterministic) part of $U_{r}^{w}$, and $\varepsilon_{r}^{w}$ is the random error part of $U_{r}^{w}$, and set $E\left(\varepsilon_{r}^{w}\right)=0$ where *E*(⋅) is the expectation operator of a random variable.

In SUE models, the stochastic travel time (also stated by (Eq 1)) is called perceived travel time since the random error term depends on every single individual's subjective perception and thus varies across the population. However, the current route travel time is an objective random variable, of which the distribution of the error term is assumed to be statistically estimated or fitted from the long collected travel time data and unvarying across travelers. Therefore, despite the identical appearance, the stochastic path travel time herein and that in SUE models have significantly different physical and economic implication.

Subsequently, by redefining the route choice probability function in the SUE models, we introduce the concept of route dominant probability, i.e.,

$$P_{r}^{w}=\text{Pr}\left(U_{r}^{w}\leq U_{i}^{w},i\in R^{w},i\neq r\right)\;\forall r\in R^{w},w\in W,$$ (2)

where Pr(⋅) is the probability operator of a random event, and $P_{r}^{w}$ is the dominant probability of path *r* in OD pair *w*.

The above route dominant probability is to give a quantitative estimation on the possibility of a route dominating the others between the same OD pair. In the SUE models, it is directly assumed to equal to the subjective choice probability, which is only applicable and available to a traveler him/herself. However, the dominant probability here is just an objective and all-known estimation on the performance of a candidate route, and the final route choice decision is made upon the overall dominant probability distribution rather than a single one. In addition, we do not require a well-designed route set *R*^*w*^(*w* ∈ *W*) as the SUE models do, since it may not be necessarily relevant to the route choice result. The irrelevance will be examined in Section 3.

Given a traffic network with stochastic travel time, the route dominant probability obviously satisfies the following relationship.

$$0\leq P_{r}^{w}\leq 1\;\forall r\in R^{w},w\in W;\;\sum_{r\in R^{w}}P_{r}^{w}=1\;\forall w\in W.$$ (3)

At SUE, travel demands will be assigned to each route in *R*^*w*^(*w* ∈ *W*) by the fraction of dominant probability. Considering that the equilibrium dominant probabilities are not necessarily equal under this assignment mechanism, there objectively exist some dominant paths with a larger dominant probability. Once the objective travel time distribution is known to all, travelers may correct their perception and reroute to achieve higher dominance. This type of individually selfish routing behavior will collectively drive a traffic network to a stationary state, which is currently defined by PdUE.

**Definition 1 (Probability-dominant user equilibrium, PdUE)**. At PdUE, given that the travel demand is fixed, all the used paths connecting the same OD pair have the maximum dominant probability; mathematically, it can be equivalently stated as

$$P_{r}^{w}\left\{ \begin{array}{r} =P_{pue}^{w}\text{, if }f_{r}^{w}>0\\ \leq P_{pue}^{w}\text{, if }f_{r}^{w}=0 \end{array}\right.\;\forall r\in R^{w},w\in W.$$ (4)

Including the feasibility conditions of path flow, the PdUE conditions can be summarized as

$$f_{r}^{w}\left(P_{pue}^{w}-P_{r}^{w}\right)=0,P_{pue}^{w}-P_{r}^{w}\geq 0,P_{pue}^{w}\geq 0\;\forall r\in R^{w},w\in W$$ (5)

and

$$\sum_{r\in R^{w}}f_{r}^{w}=q^{w}\;\forall w\in W;\;f_{r}^{w}\geq 0\;\forall r\in R^{w},w\in W,$$ (6)

where $f_{r}^{w}$ is flow on path *r* in OD pair *w*, $P_{pue}^{w}$ is the largest dominant probability of the routes between OD pair *w*, and *q*^*w*^ is the travel demand in OD pair *w*.

According to Definition 1, the routes with smaller dominant probabilities will not be selected at PdUE, implying that the summation of dominant probabilities over the routes between an OD pair may be less than one. It is known that, for SUE, this probability summation is definitely equal to one, and the SUE flow pattern varies over different route set, which makes the definition of route set important and sensitive. The dominant probability distributions at PdUE vary against different route sets; however, PdUE can still be immobile when the difference between two route sets is exactly in the unused routes. The logit PdUE to be discussed in Section 3 offers evidence to this conjecture.

Let row vector $P\left(\cdot \right)=\left(P_{r}^{w}|r\in R^{w},w\in W\right)$, column vector $f={\left(f_{r}^{w}|r\in R^{w},w\in W\right)}^{T}$, and Ω defined by (Eq 6) be the set of feasible path flow. We give an equivalent reformulation for PdUE as follows.

**Proposition 1**. PdUE solves the following varitional inequality problem (VIP), i.e.,

$$P\left(f^{*}\right)\left(f-f^{*}\right)\leq 0\;\forall f\in \Omega .$$ (7)

**Proof**. It is easy to concluded that the flow f* solves VIP (7) if and only if it solves the following linear programming

$${\text{max}}_{f\in \Omega }P\left(f^{*}\right)f.$$ (8)

Considering that the dominant probability of a route is non-negative, we can conclude that the primal-dual optimality conditions of above linear programming are exactly Eqs (5) and (6). Proof completed.

Subsequently, we give two propositions associated with PdUE.

**Proposition 2**. The deterministic user equilibrium[20] is a special case of PdUE.

**Proof**. According to the definition of deterministic user equilibrium, all used paths within the same OD pair have the equally minimum travel cost. In the framework of PdUE, a deterministic traffic network implies that the error term of stochastic travel time in (Eq 1) is absent. Thus, the dominant probability of a route can only be 0 or 1. If it is 0, this route cannot be the least-costly one, and vice versa; likewise, if it is 1, this route must be the least-costly one, and vice versa. In short, the dominant probability of a route is 1 if and only if it is the least-costly route between its own OD pair. It follows from PdUE that all used paths have the largest dominant probabilities, which are exactly the least-costly routes within their own OD pair. Therefore, the deterministic user equilibrium is a special case of PdUE. Proof completed.

**Proposition 3**. At PdUE, if all the routes considered in the route set between an OD pair are used, and then these routes equally split the overall dominant probability; mathematically, it means that

$$f_{r}^{w}>0\;\forall r\in R^{w},w\in W\Rightarrow P_{r}^{w}=\frac{1}{\left|R^{w}\right|}\;\forall r\in R^{w},w\in W,$$ (9)

where |*R*^*w*^| denotes the number of routes contained in set *R*^*w*^.

**Proof**. According to Definition 1, all those used routes at PdUE between the same OD pair have the same dominant probability. Recalling (Eq 3), we have $P_{r}^{w}=\frac{1}{\left|R^{w}\right|}\;\forall r\in R^{w},w\in W$. Proof completed.

Proposition 3 provides a direct and convenient method to identify the PdUE dominant probability distribution against some special cases. However, note that it does not mean that the corresponding path flows are equal.

### 2.2 A behavioural dynamic description

In this subsection, a probability-dominant rerouting dynamic (PdRD) model is developed to describe the dominance-seeking rerouting behavior in a non-equilibrium stochastic network. Due to the focus of this study, we do not detail the rerouting models here, and the readers are referred to, e.g., Ref. [21], for a more detailed literature review on this topic. Note that pursuing the largest dominance does not mean that all travelers who are using the less dominant routes will reroute to the largest dominant route in the next time, because some (if not all) travelers will consider the post-outcome from synchronously piping into a single route. Hence, it is reasonable to assume for the present PdRD that just a fraction of travelers finally reroute. Prior to formulate PdRD, we define

$$P_{*}^{wt}=\text{max}\left\{P_{r}^{wt}|r\in R^{w}\right\}\text{ and }R_{*}^{wt}=\left\{r\in R^{w}|P_{r}^{wt}=P_{*}^{wt}\right\}\;\forall w\in W,t\geq 0,$$ (10)

where *t* is the time index, $P_{*}^{wt}$ denotes the largest route dominant probability in OD pair *w* at time *t*, and $R_{*}^{wt}$ is the set of the largest dominant routes in OD pair *w* at time *t*.

Let $R_{-}^{wt}$ be the complementary set of $R_{*}^{wt}$ under the universal set *R*^*w*^, $\left|R_{*}^{wt}\right|$ be the number of paths included in set $R_{*}^{wt}$, and $f_{r}^{wt}$ be the flow on route *r* in OD pair *w* at time *t*. PdRD is formulated as follows:

$$f_{r}^{w(t+1)}-f_{r}^{wt}=\left\{ \begin{array}{r} \frac{1}{\left|R_{*}^{wt}\right|}\sum_{i\in R_{-}^{wt}}f_{i}^{wt}\rho_{i}^{wt}\text{, if }r\in R_{*}^{wt}\\ -f_{r}^{wt}\rho_{r}^{wt}\text{, if }r\in R_{-}^{wt} \end{array}\right.\;\forall r\in R^{w},w\in W,t\geq 0$$ (11)

where

$$\rho_{r}^{wt}=1-\text{exp}\left(-\theta^{w}\left(P_{*}^{wt}-P_{r}^{wt}\right)\right).$$ (12)

In Eqs (11) and (12), *θ*^*w*^ is a positive parameter reflecting the travelers’ reaction degree in OD pair *w*, $\rho_{r}^{wt}$ is the rerouting protocol to estimate the fraction of flow that swaps off route *r* in OD pair *w*, and it grows nonlinearly with the increase of probability difference.

It can be seen from (Eq 11) that (i) traffic uniaxially swaps from the less dominant routes (contained in set $R_{-}^{wt}$) to the most dominant ones (contained in $R_{*}^{wt}$); (ii) if the most dominant route is not unique, all routes in $R_{*}^{wt}$ will share the swap-in flow equally. This equally-sharing idea was initially introduced in our previous works[21,22] that deal with day-to-day traffic evolution in a deterministic network.

Next, we examine several critical logical and behavioral properties associated with PdRD.

**Property 1 (Non-over-swapping)**. For PdRD, during every path-swapping process, the total swap-off flow from a path cannot spill its initial value, mathematically, i.e.,

$$0\leq \rho_{r}^{wt}\leq 1\;\forall r,w,t.$$ (13)

**Proof**. Since $1-\text{exp}\left(-\theta^{w}\left(P_{*}^{wt}-P_{r}^{wt}\right)\right)\leq \text{1}$, we have $0\leq \rho_{r}^{wt}\leq 1\;\forall r,w,t$. Then, Property 1 is proved. Proof completed.

**Property 2**. According to PdRD, we have $\rho_{r}^{wt}>0$ if $r\in R_{-}^{wt}$, and $\rho_{r}^{wt}=0$ if $r\in R_{*}^{wt}$.

**Proof**. From (Eq 11), it follows that $P_{r}^{wt}<P_{*}^{wt}$ if $r\in R_{-}^{wt}$, and $P_{r}^{wt}=P_{*}^{wt}$ if $r\in R_{*}^{wt}$; further $1-\text{exp}\left(-\theta^{w}\left(P_{*}^{wt}-P_{r}^{wt}\right)\right)>0$ if $r\in R_{-}^{wt}$, and $1-\text{exp}\left(-\theta^{w}\left(P_{*}^{wt}-P_{r}^{wt}\right)\right)=0$ if $r\in R_{*}^{wt}$. Recalling (Eq 12), Property 2 holds. Proof completed.

**Property 3 (Solution invariance)**. For PdRD, if the initial flow pattern is feasible, so do the remaining updated flow patterns.

**Proof**. Given a feasible initial flow pattern, i.e.,

$$\sum_{r}f_{r}^{w0}=q^{w}\;\forall w\text{; }f_{r}^{w0}\geq 0\;\forall r,w,$$ (14)

Property 3 requires that the remaining updated path flows retain non-negativity and travel demands retain conservation, i.e.,

$$\sum_{r}f_{r}^{wt}=q^{w}\;\forall w,t;\;f_{r}^{wt}\geq 0\;\forall r,w,t.$$ (15)

**Non-negativity**. From Property 1 and (Eq 11), we can conclude that

$$f_{r}^{w(t+1)}=\left\{ \begin{array}{r} f_{r}^{wt}+\frac{1}{\left|R_{*}^{wt}\right|}\sum_{i\in R_{-}^{wt}}f_{i}^{wt}\rho_{i}^{wt}\geq 0,\text{ if }r\in R_{*}^{wt}\\ f_{r}^{wt}-f_{r}^{wt}\rho_{r}^{wt}=f_{r}^{wt}\left(1-\rho_{r}^{wt}\right)\geq 0,\text{ if }r\in R_{-}^{wt} \end{array}\right.$$ (16)

Hence, we have $f_{r}^{wt}\geq 0\;\forall t\geq 1$ if $f_{r}^{w0}\geq 0$, i.e., non-negativity is satisfied.

**Conservation**. According to (Eq 11), we have

$$\begin{array}{ll} \sum_{r\in R^{wt}}f_{r}^{w(t+1)} & =\sum_{r\in R_{*}^{wt}}f_{k}^{wt}+\sum_{r\in R_{*}^{wt}}\sum_{i\in R_{-}^{wt}}\frac{1}{\left|R_{*}^{wt}\right|}f_{i}^{wt}\rho_{i}^{wt}+\sum_{r\in R_{-}^{wt}}f_{r}^{wt}-\sum_{k\in R_{-}^{wt}}f_{r}^{wt}\rho_{r}^{wt}\\ & =\sum_{r\in R^{wt}}f_{r}^{wt}+\sum_{i\in R_{-}^{wt}}f_{i}^{wt}\rho_{i}^{wt}\sum_{r\in R_{*}^{wt}}\frac{1}{\left|R_{*}^{wt}\right|}-\sum_{r\in R_{-}^{wt}}f_{r}^{wt}\rho_{r}^{wt}\\ & =\sum_{r\in R^{wt}}f_{r}^{wt}+\sum_{i\in R_{-}^{wt}}f_{i}^{wt}\rho_{i}^{wt}-\sum_{r\in R_{-}^{wt}}f_{r}^{wt}\rho_{r}^{wt}\\ & =\sum_{r\in R^{wt}}f_{r}^{wt}=\cdots =\sum_{r\in R^{wt}}f_{r}^{w0}=q^{w}. \end{array}$$ (17)

Thus, the conservation property holds. Proof completed.

Besides behavioral intuitiveness, Properties 1 and 3 additionally show that PdRD is logically sound. Subsequently, we examine whether PdUE is the stationary state of PdRD. To this end, we first introduce the definition of PdRD's stationary path flow pattern as follows.

**Definition 2 (Stationary path flow pattern)**. PdRD’s stationary path flow pattern is a set of network path flow states, and starting from anyone of them, PdRD reproduces them.

**Proposition 4**. For PdRD, if $f_{r}^{w(t+1)}=f_{r}^{wt}\;\forall r,w,t$, then vector $f^{t}=\left(f_{r}^{wt}|r\in R^{w},w\in W\right)$ is a stationary path flow pattern, and vice versa.

**Proof**. Since PdRD is a determined one-to-one dynamic process (i.e., PdRD produces a single output, given a determined input of flow state), the sufficiency of Proposition 4 can be proved by recurrence. The necessity can be directly concluded from Definition 2. Proof completed.

We now give the relationship between PdRD's stationary path flow pattern and PdUE as follows.

**Proposition 5**. PdRD’s stationary path flow pattern is equivalent to PdUE.

**Proof**. Based on Proposition 4, to prove Proposition 5, we only need to prove that vector **f**^**t**^ with $f_{r}^{w(t+1)}=f_{r}^{wt}\;\forall r,w,t$ is equivalent to PdUE.

**Sufficiency**. When $f_{r}^{w(t+1)}=f_{r}^{wt}\;\forall r,w,t$, according to (Eq 12), we can conclude that

$$\left\{ \begin{array}{r} \frac{1}{\left|R_{*}^{wt}\right|}\sum_{i\in R_{-}^{wt}}f_{i}^{wt}\rho_{i}^{wt}=0,\text{ if }r\in R_{*}^{wt}\\ f_{r}^{wt}\rho_{r}^{wt}=0,\text{ if }r\in R_{-}^{wt} \end{array}\right.$$ (18)

Recalling Property 2, we have $f_{r}^{wt}=0$ if $r\in R_{-}^{wt}$, i.e., $f_{r}^{wt}=0$ if $P_{r}^{wt}<P_{*}^{wt}$, which further yields that $P_{r}^{wt}=P_{*}^{wt}$ if $f_{r}^{wt}>0$. We can also conclude that $P_{r}^{wt}\leq P_{*}^{wt}$ if $f_{r}^{wt}\geq 0$ from $P_{r}^{wt}\leq P_{*}^{wt}\;\forall r\in R^{w}$, deducing $P_{r}^{wt}\leq P_{*}^{wt}$ if $f_{r}^{wt}=0$.

**Necessity**. Suppose that **f**^**t**^ is at PdUE, and then we have $P_{r}^{wt}=P_{*}^{wt}$ if $f_{r}^{wt}>0$ and $P_{r}^{wt}\leq P_{*}^{wt}$ if $f_{r}^{wt}=0$, meaning that traffic solely distributes on the largest dominant paths and there is no traffic on the less dominant ones, i.e., $f_{r}^{wt}=0$ if $r\in R_{-}^{wt}$, further giving $\sum_{i\in R_{-}^{wt}}f_{i}^{wt}\rho_{i}^{wt}=0$. Hence, according to (Eq 11), we have $f_{r}^{w(t+1)}=f_{r}^{wt}\;\forall r,w,t$.

According to the above proofs, Proposition 6 holds. Proof completed.

Proposition 5 verifies that PdRD is capable of intuitively explaining the behavioral dynamic mechanism of PdUE, which in turn implies that PdUE is exactly the equilibrium state resulted from the dominance-seeking non-corporative routing game.

## 3. Logit formula of PdUE

Section 2 gives a general modeling framework of PdUE. Considering the popularity of the logit model[23] among the discrete choice models and to facilitate the application of PdUE, this section elaborates on the logit reformulation of PdUE. To this end, let $\varepsilon_{r}^{w}$ (*r* ∈ *R*^*w*^, *w* ∈ *W*) in (Eq 1) be the independently and identically distributed Gumbel variates, and then the dominant probability of (Eq 2) can be expressed in a closed form as

$$P_{r}^{w}=\frac{\text{exp}\left(-C_{r}^{w}\right)}{\sum_{i\in R^{w}}\text{exp}\left(-C_{i}^{w}\right)}\;\forall r\in R^{w},w\in W,$$ (19)

where exp(⋅) is the exponential operator of Euler's constant.

For the derivation process of (Eq 19), one can refer to Ref. [24]. Here, we present the formula of the logit-based PdUE (Logit-PdUE) as follows.

**Proposition 6**. At Logit-PdUE, all the used paths between the same OD pair have equal and minimum measurable travel time, mathematically, i.e.,

$$C_{r}^{w}\left\{ \begin{array}{r} =\pi^{w}\text{, if }f_{r}^{w}>0\\ \geq \pi^{w}\text{, if }f_{r}^{w}=0 \end{array}\right.\;\forall r\in R^{w},w\in W,$$ (20)

where $\pi^{w}=\text{min}\left\{C_{r}^{w}|r\in R^{w},w\in W\right\}$. Including the feasibility conditions of path flow, the Logit-PdUE conditions can be summarized as (Eq 6) and

$$f_{r}^{w}\left(C_{r}^{w}-\pi^{w}\right)=0,C_{r}^{w}-\pi^{w}\geq 0,\pi^{w}\geq 0\;\forall r\in R^{w},w\in W.$$ (21)

**Proof**. According to the definition of PdUE and supposing that path *r* and path *l* in *R*^*w*^ are used at PdUE, we can conclude that $P_{r}^{w}=P_{l}^{w}=\text{max}\left\{P_{i}^{w}|i\in R^{w}\right\}$. Recalling the logit formula of (Eq 19), it implies that $\frac{\text{exp}\left(-C_{r}^{w}\right)}{\sum_{i\in R^{w}}\text{exp}\left(-C_{i}^{w}\right)}=\frac{\text{exp}\left(-C_{l}^{w}\right)}{\sum_{i\in R^{w}}\text{exp}\left(-C_{i}^{w}\right)}$ and further $\text{exp}\left(-C_{r}^{w}\right)=\text{exp}\left(-C_{l}^{w}\right)$. Since exp(⋅) is a strictly monotonically increasing function, we can conclude $C_{r}^{w}=C_{l}^{w}=\pi^{w}=\text{min}\left\{C_{r}^{w}|r\in R^{w},w\in W\right\}$; in other words, all the used paths between the same OD pair have equal and minimum measurable travel time at Logit-PdUE. Proof completed.

Proposition 6 shows that, in comparison with the general formulation of PdUE given in (Eq 5), Logit-PdUE has significantly simplified the formulation, and leads to an elegant and familiar mathematical structure that can readily solved by the deterministic user equilibrium algorithms. Next, we state an equivalent VIP formulation of Logit-PdUE in Proposition 7.

**Proposition 7**. Logit-PdUE solves the following VIP, i.e.,

$$C\left(f^{*}\right)\left(f-f^{*}\right)\geq 0\;\forall f\in \Omega ,$$ (22)

where $C\left(\cdot \right)=\left(C_{r}^{w}|r\in R^{w},w\in W\right)$.

**Proof**. It is easy to concluded that the flow f* solves VIP (22) if and only if it solves the following linear programming

$${\text{min}}_{f\in \Omega }C\left(f^{*}\right)f.$$ (23)

Since the measurable travel time of a route is non-negative, we can conclude that the primal-dual optimality conditions of above linear programming are exactly Eqs (6) and (21). Accordingly, Logit-PdUE solves VIP (22). Proof completed.

Then, we examine the existence and uniqueness of Logit-PdUE.

**Proposition 8**. VIP (22) has at least one solution if **C**(**f**) is continuous with respect to **f**, and has a unique solution if **C**(**f**) is strictly monotonely increasing with respect to **f**.

**Proof**. What Proposition 8 stated is the basic property of a VIP problem like VIP (22). Thus, the readers are referred to Ref. [25] for a complete proof. For brevity, we do not state it here again. Proof completed.

It is known that the continuity assumption in Proposition 8 is usually valid, whereas the monotonicity requirement is usually difficult to meet/satisfy. Therefore, in general, Logit-PdUE is existent but may not be unique.

Obviously, the deterministic user equilibrium is also a special case of Logit-PdUE, which coincides with the conclusion of Proposition 2. In addition, let $K_{*}^{w}$ denote the set of routes used at Logit-PdUE and according to Propositions 6 and 7, it is easy to conclude that, for any route set $Z^{w}\left(K_{*}^{w}\subseteq Z^{w}\subseteq K^{w}\right)$, Logit-PdUE will not change; in other words, Logit-PdUE does not depend on a specific route set. Thus, it is unnecessary for Logit-PdUE to take many efforts to construct a well-designed route set as SUE has to.

## 4. Experimental analysis

As previously analyzed, when the probability distribution of route travel time is available to travelers, a stochastic traffic network will finally reach PdUE, of which the underlying routing strategy is called as a probability-dominant strategy (PDS). However, note that travelers can still take a SUE-like routing strategy (i.e., selecting a route according to the dominant probability) even if the travel time distribution is known to them. Such a SUE-like routing strategy is called as a dominant probability strategy (DPS). In this section, a hypothetical experiment is designed to discuss PDS and DPS from an individual selfish perspective. More precisely, it is to determine that which routing strategy (between PDS and DPS) is better for an individual traveler when the objective stochastic travel time distribution is available to him/her.

### 4.1 Experiment scenario

**Fig 1:**
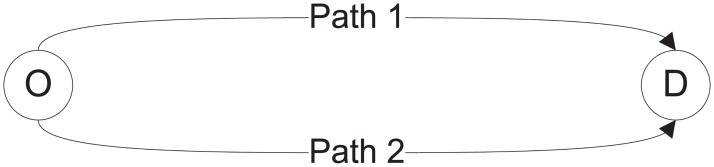
The experiment network.

**Initial condition**: Consider a simple network (see Fig 1) with a single OD pair connected by two paths (i.e., Paths 1 and 2); and assume that the travel demand is very large and the probability distribution of route travel time is initially unavailable. Thus, travelers select routes according to the personal perception. When the network reaches SUE, the choice probabilities (or dominant probabilities) of Paths 1 and 2 are *p* and *q* (p > *q* and *p* + *q* = 1), respectively.

**Question**: Imagining that a traveler confronts *N* trips in this network within a period of time. Provided that the objective path travel time distribution is now available to him/her, will he/she insist on *N* times of DPS or turn to *N* times of PDS?

To answer this question, the essence is to determine which choice gives rise to more utility. For a rational person, it is intuitive to assume that a larger utility implies achieving more times of successes (i.e., experiencing less travel time). In this experiment, selecting PDS implies assigning *N* times to Path 1, and selecting DPS implies assigning *pN* times to Path 1 and *qN* times to Path 2. Since the travel demand is very large and only this traveler knows the objective travel time distribution information, it is reasonable to assume that the SUE probability distribution changes little because of a single traveler’s route choice. Then, we can obtain the probabilities of achieving *n* times of successes within the *N* trips for two routing strategies as follows.

For PDS:

$$\text{Pr}\left(n\right)=C_{N}^{n}p^{n}q^{N-n}.$$ (24)

For DPS:

$$\text{Pr}\left(n\right)=\sum_{i=0}^{n}\left[\left(C_{pN}^{t}p^{i}q^{pN-i}\right)\cdot \left(C_{qN}^{n-i}q^{n-i}p^{qN-(n-i)}\right)\right].$$ (25)

Based on Eqs (24) and (25), we can obtain the cumulative probabilities with respect to two routing strategies as follows.

For PDS:

$$\text{Pr}(n\geq m)=\sum_{n=m}^{N}C_{N}^{n}p^{n}q^{N-n}.$$ (26)

For DPS:

$$\text{Pr}(n\geq m)=\sum_{n=m}^{N}\sum_{i=0}^{n}\left[\left(C_{pN}^{t}p^{i}q^{pN-i}\right)\cdot \left(C_{qN}^{n-i}q^{n-i}p^{qN-(n-i)}\right)\right].$$ (27)

### 4.2 Discussion

First, we discuss the dominant relationship between two routing strategies in two extreme cases, i.e., the best case to achieve full success (i.e., N times of successes) and the worst case to achieve no success. Table 1 gives the probabilities with respect to two extreme cases.

**Table 1:** The probability distribution of success in two extreme cases.

Case	Probability of success		Probability difference (PDS−DPS)
	PDS	DPS	
No success	*q*^*N*^	*p*^*qN*^*q*^*pN*^	Negative
Full success	*p*^*N*^	*p*^*pN*^*q*^*qN*^	Positive

Table 1 indicates that: (i) PDS yields a smaller probability than DPS to encounter 0 success, meaning that PDS is more robust; (ii) PDS yields a larger probability than DPS to achieve *N* times of successes, implying that PDS yields a better optimality. Accordingly, it can be conclude that, whether out of insurance or striving for perfection, PDS is a superior choice to DPS.

Next, we discuss the dominance between two routing strategies in general cases. To this end, the stochastic dominance theory (refer to Ref. [26] for more detail), which is developed to rank two stochastic alternatives, is applied. Among the three stochastic dominance principles, the first-order dominance principle (FDP) is the most general one since it makes the weakest assumption, i.e., the perceived utility grows with the increase of positive outcome. As mentioned above, the utility in present experiment is calculated by the times of successes, and a larger utility implies more times of successes. Then, it follows from FDP that DPS is dominated by PDS if and only if the probability of DPS achieving more than *m* (∀*m* ∈ [0, *N*]) times of successes is smaller than that of PDS. Based on Eqs (26) and (27), it means that

$$\sum_{n=m}^{N}\left\{\sum_{i=0}^{n}\left[\left(C_{pN}^{t}p^{i}q^{pN-i}\right)\cdot \left(C_{qN}^{n-i}q^{n-i}p^{qN-(n-i)}\right)\right]-C_{N}^{n}p^{n}q^{N-n}\right\}\leq 0\;\forall m\in [0,N].$$ (28)

(Eq 28) claims that the probability of DPS achieving more than any times of successes is consistently smaller than that of PDS. According to FDP, DPS is dominated by PDS if and only if (Eq 28) holds. Table 1 already proves that (Eq 28) holds for *m* ∈ {0, 1, *N*}. Due to the difficulty in proving that (Eq 28) holds for the other cases, currently we just make a conjecture. Future works are expected to offer an effective proof.

**Conjecture 1**. For above experiment, DPS is dominated by PDS.

**Fig 2:**
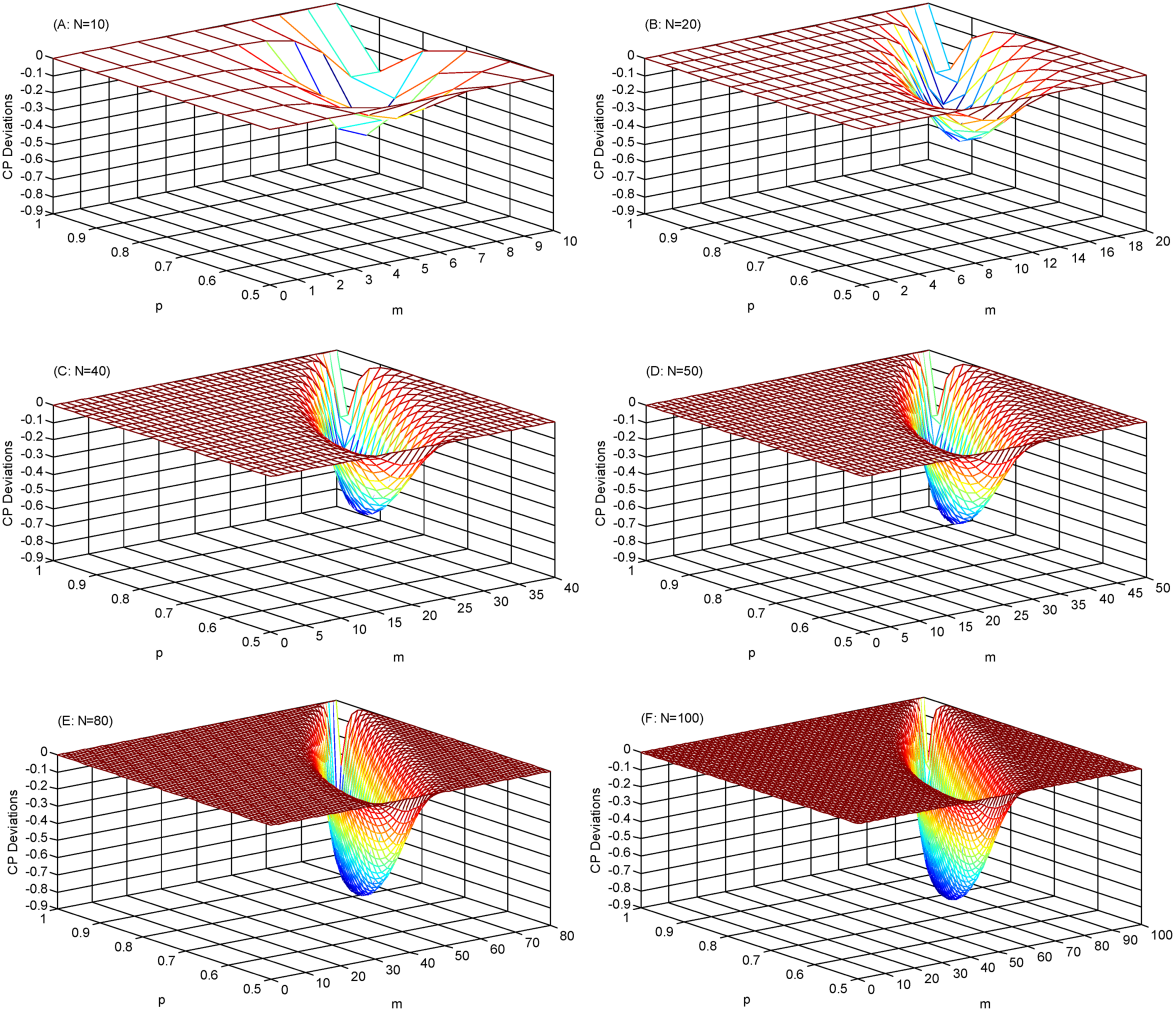
The numerical testing results under different *N*s. Figs 2A–2F display the testing results when N are 10, 20, 40, 50, 80 and 100, respectively. The Z-axis title “CP Deviation”estimates the value on the left side of (Eq 28).

We demonstrate the conjecture in a limited number of numerical test results for (Eq 28); see Fig 2. Due to the capacity limitation in combination computation, here we only present the results when *N* = 10~100.

It can be seen from Fig 2 that the CP Deviations are uniformly less than zero, demonstrating that (Eq 28) holds for the above cases. More precisely, it means that, besides *m* ∈ {0, 1, *N*}, DPS is also dominated by PDS in above cases. In addition, Fig 2 still indicates that the minimum CP Deviations decreases steadily as N grows, implying that the superiority of PDS is more significant under more trips.

Taken Table 1 and Fig 2 together, it can be concluded that PDS is at least not inferior to DPS and in above (maybe all) cases superior to DPS for individuals. Now, we can answer PDS to the question made in previous, i.e., taking PDS in the next *N* times of trips. Note that in this routing experiment this traveler is the same as the others except having the probability distribution information of route travel time. Therefore, if another one also has the information, he/she will take PDS as well. When everyone has the information, those who are using the less dominant routes will partly reroute to achieve (possibly) more dominance. The collective rerouting behavior can be captured by the PdRD in Section 2.2, of which the stationary state is exactly PdUE. Accordingly, PdUE is a self-driven desirable equilibrium state in a stochastic traffic network when the travel time distribution is available to all.

## 5. Conclusions and future works

This paper proposes a PdUE model to describe the selfish routing equilibrium in a stochastic traffic network where the travel time distribution is assumed to be available to travelers. Different from the existing SUE and RUE, at PdUE, the OD demand is only assigned to the routes with the largest dominant probability. The definition and the general formulation of PdUE are made. An intuitive dynamic model, i.e., PdRD, is developed to explain the behavioral mechanism of PdUE. It is proved that the stationary state of PdRD is PdUE. Thus, PdUE is the resulting equilibrium state from the dominance-seeking non-corporative routing game. To facilitate the application, the logit formula of PdUE is developed, of which a well-designed route set is not indispensable and the equivalent varitional inequality formation is simple. Given the travel time distribution and conducted by the dominance theory, the SUE and PdUE routing strategies (i.e., DPS and PDS, respectively) are discussed through a hypothetical routing experiment from an individual selfish perspective. It is found that, whether out of insurance or striving for perfection, PDS is a superior choice to DPS. For more general cases, the conducted numerical tests show the same conclusion. Therefore, PdUE (rather than the conventional SUE) is a desirable equilibrium in a stochastic traffic network when the probability distributions of travel time are available.

For the future study direction, it is worthy of developing an analytical proof for Conjecture 1. In addition, by relaxing the independent and identical distribution assumption with respect to the random error terms in stochastic travel time, the present Logit-PdUE model can be accordingly extended to many other versions.
